# 

**Published:** 1860-01-01

**Authors:** 


### THE JOURNAL

OF

### PSYCHOLOGICAL MEDICINE

AND

### MENTAL PATHOLOGY.

EDITED BY

FORBES WINSLOW, M.D., D.C.L. OXON.

VOL. XIII.

Surgeon Genl's Office
LIBRARY
22057
Washington, D.C.

LONDON:
JOHN CHURCHILL, NEW BURLINGTON STREET.
MDCCCLX.

WI
J0857N

LONDON:

SAVILL AND EDWARDS, PRINTERS, CHANDOS STREET,

COVENT GARDEN.

*In the press, and will be published early in February*
*One Vol., Octavo,* 600 *pages.*

A TREATISE

ON

### OBSCURE DISEASES OF THE BRAIN AND MIND:

THEIR INCIPIENT SYMPTOMS, PATHOLOGY, TREATMENT, AND PROPHYLAXIS.

By FORBES WINSLOW, M.D.
HON. D.C.L. OXON.

ORDERS FOR THIS WORK ARE TO BE TRANSMITTED TO

JOHN CHURCHILL, PUBLISHER, NEW BURLINGTON STREET,

LONDON.

